# Development of a Gene and Nucleic Acid Delivery System for Skeletal Muscle Administration via Limb Perfusion Using Nanobubbles and Ultrasound

**DOI:** 10.3390/pharmaceutics15061665

**Published:** 2023-06-06

**Authors:** Shohko Sekine, Sayaka Mayama, Nobuaki Nishijima, Takuo Kojima, Yoko Endo-Takahashi, Yuko Ishii, Hitomi Shiono, Saki Akiyama, Akane Sakurai, Sanae Sashida, Nobuhito Hamano, Rui Tada, Ryo Suzuki, Kazuo Maruyama, Yoichi Negishi

**Affiliations:** 1 Department of Drug Delivery and Molecular Biopharmaceutics, School of Pharmacy, Tokyo University of Pharmacy and Life Sciences, Tokyo 192-0392, Japanendo@toyaku.ac.jp (Y.E.-T.); nhamano@toyaku.ac.jp (N.H.); ruitada@toyaku.ac.jp (R.T.); 2 Laboratory of Drug and Gene Delivery Research, Faculty of Pharma-Sciences, Teikyo University, 2-11-1 Kaga, Itabashi-ku, Tokyo 173-8605, Japan; r-suzuki@pharm.teikyo-u.ac.jp; 3 Advanced Comprehensive Research Organization (ACRO), Teikyo University, Tokyo 173-8605, Japan; maruyama@pharm.teikyo-u.ac.jp; 4 Laboratory of Ultrasound Theranostics, Faculty of Pharma-Sciences, Teikyo University, Tokyo 173-8605, Japan

**Keywords:** PMO delivery, ultrasound, nanobubble, limb vein injection, Duchenne muscular dystrophy (DMD)

## Abstract

Strategies for gene and nucleic acid delivery to skeletal muscles have been extensively explored to treat Duchenne muscular dystrophy (DMD) and other neuromuscular diseases. Of these, effective intravascular delivery of naked plasmid DNA (pDNA) and nucleic acids into muscles is an attractive approach, given the high capillary density in close contact with myofibers. We developed lipid-based nanobubbles (NBs) using polyethylene-glycol-modified liposomes and an echo-contrast gas and found that these NBs could improve tissue permeability by ultrasound (US)-induced cavitation. Herein, we delivered naked pDNA or antisense phosphorodiamidate morpholino oligomers (PMOs) into the regional hindlimb muscle via limb perfusion using NBs and US exposure. pDNA encoding the luciferase gene was injected with NBs via limb perfusion into normal mice with application of US. High luciferase activity was achieved in a wide area of the limb muscle. DMD model mice were administered PMOs, designed to skip the mutated exon 23 of the dystrophin gene, with NBs via intravenous limb perfusion, followed by US exposure. The number of dystrophin-positive fibers increased in the muscles of mdx mice. Combining NBs and US exposure, which can be widely delivered to the hind limb muscles via the limb vein, could be an effective therapeutic approach for DMD and other neuromuscular disorders.

## 1. Introduction

Duchenne muscular dystrophy (DMD) is considered the most common serious form of muscular dystrophy; it is attributed to nonsense or frame-shift mutations in the X chromosome DMD gene encoding the dystrophin protein that leads to loss of expression and a lethal muscle-wasting disorder, which is estimated to impact approximately 1/3500–5000 male births worldwide [1,2]. Conversely, milder Becker muscular dystrophy is closely associated with mutations that cause internal deletions and partially functional dystrophins while maintaining the reading frame [3,4]. One therapeutic strategy for treating DMD involves the application of antisense oligonucleotides (ASOs) to induce exon skipping of dystrophin genes, thereby eliminating the disease-inducing mutation and restoring the reading frame of the gene transcript [5,6,7,8,9].

Recently, exon-skipping ASOs (Exondys 51, Vyondys 53, and Viltepso Amondys 45) approved by the United States Food and Drug Administration (FDA) have been used clinically for exon skipping of DMD genes [10]. The ASO backbone utilizes phosphorodiamidate morpholino oligomers (PMOs). However, patients with DMD who were treated with exon-skipping drugs had markedly low muscle levels of restored dystrophin expression, approximately 1% of the levels detected in healthy controls [11,12].

PMOs exhibit superior nuclease stability and safety when compared with other ASO chemical compounds [13]. However, owing to their uncharged backbone and low protein-binding affinity, PMOs undergo rapid renal clearance from the bloodstream with poor cellular uptake, necessitating high and costly dosages. Although PMOs are currently available for clinical application, substantial obstacles need to be addressed to achieve therapeutic efficacy in patients with DMD.

Nonviral physical delivery methods [14,15,16,17] and ultrasound (US) can boost the efficiency of drug and gene delivery to tissues and cells using a sonoporation technique [15]. It has been suggested that US could enable the entry of extracellular drugs and genes into viable cells by creating transient pores in the cell membrane following ultrasonic cavitation [14,17,18,19]. Furthermore, the application of transcutaneous focused US exposure to target tissues after administering microbubbles (MBs), which act as echo-contrast gas agents for medical US imaging, can increase transient cell membrane and vasculature permeability, facilitating site-specific therapeutic drug and gene delivery by inducing local acoustic cavitation with minimal cellular damage [20,21,22,23]. To improve blood retention and reach deeper tissues, we have previously developed nanobubbles (NBs) based on polyethylene glycol (PEG)-modified liposomes and reported that the developed NBs exhibit US contrast agent and gene delivery capabilities in combination with US exposure [24,25,26,27,28,29,30].

In the present study, we developed an efficient delivery system for targeting skeletal muscles via intravenous limb injection using NBs and US. To achieve this, we first applied a tourniquet around the upper part of the lower limb. Then, luciferase-encoding pDNA was injected with NBs via the intravenous limb vein in normal mice, followed by US exposure. Next, PMO, designed to skip the mutated exon 23 of murine dystrophin, was delivered into the muscles of DMD model mice using a similar treatment strategy involving NBs and US exposure.

## 2. Materials and Methods

### 2.1. ASOs

PMO M23D (+7-8) (5′-GGCCAAACCTCGGCTTACCTGAAAT-3′) was purchased from Gene Tools (Philomath, OR, USA). The sequences were designed to anneal to the last 7 bases of exon 23 and the first 18 bases of intron 23 [9].

### 2.2. Animals

The use of animals and relevant experimental procedures was approved by the Tokyo University of Pharmacy and Life Science Committee on the Care and Use of Laboratory Animals. ICR mice were used in each plasmid DNA (pDNA) delivery experiment. C57BL/10ScSnmdx mice (mdx) with a nonsense mutation in exon 23 of dystrophin were used for each PMO delivery experiment. Normal C57BL/6 mice were used as positive controls for immunohistochemistry.

### 2.3. NB Preparation

NBs were prepared as previously described [24,25]. Briefly, we prepared PEG liposomes comprising 1,2-dipalmitoyl-sn-glycero-3-phosphocholine (DPPC) (NOF Corporation, Tokyo, Japan) and 1,2-distearoyl-sn-glycero-3-phosphatidyl-ethanolamine-polyethyleneglycol (DSPE-PEG2000-OMe) (NOF Corporation, Tokyo, Japan) in a molar ratio of 94:6 using the reverse-phase evaporation method. All reagents were dissolved in 1:1 (*v*/*v*) chloroform/diisopropyl ether. Phosphate-buffered saline was added to the lipid solution and the mixture was sonicated, followed by evaporation at 47 °C. The organic solvent was completely removed, and the liposome size was adjusted to less than 200 nm using extruding equipment and a sizing filter (pore size: 200 nm) (Nuclepore Track-Etch Membrane, Cytiva, MA, USA). The lipid concentrations were determined using the phospholipid C test (Wako Pure Chemical Industries, Ltd., Osaka, Japan). NBs were prepared using liposomes and perfluoropropane gas (Takachio Chemical Ind. Co., Ltd., Tokyo, Japan). First, a 2 mL sterile vial containing 0.8 mL liposome suspension (lipid concentration:1 mg/mL) was pre-filled with perfluoropropane gas, capped, and pressurized with an additional 3 mL of perfluoropropane gas. The vial was placed in a bath-type sonicator (42 kHz, 100 W) (BRANSONIC 2510j-DTH; Branson Ultrasonics Co., Danbury, CT, USA) for 5 min to form NBs. The zeta potential and mean diameter of the NBs were measured using the light-scattering method with a zeta potential/particle sizer (Nicomp 380ZLS; Santa Barbara, CA, USA). Herein, NBs (particle size: approximately 500 nm) were used as described previously [28].

### 2.4. Intravenous or Intramuscular Delivery of Luciferase pDNA Vectors into the Muscle

Briefly, ICR mice (5–6 weeks old, male) were anesthetized, followed by hair removal at the hindlimb site. Prior to each injection, a tourniquet was placed around the upper hind limb to restrict blood flow across the mouse limb. Subsequently, a mixture of pDNA (pcDNA3-Luc; 30 mg/15 mL) and NBs (10 mg/10 mL) was administered intravenously through the great saphenous vein using a syringe pump (KDS 100: KD Scientific Inc., Holliston, MA, USA) at a constant flow rate (25 mL/144 s). Immediately after the injection, US (frequency, 1 MHz; duty, 50%; intensity, 0.1, 0.5, 1, 2, or 3 W/cm^2^; time, 120 s) was applied to the hamstring muscle. We used a Sonitron 2000 (NEPA GENE Co., Ltd., Chiba, Japan) as the US generator. The mice were euthanized five days post-injection, and the limb muscles were harvested and separated into five groups (quadriceps, biceps, hamstring, gastrocnemius, and tibialis). Tissue homogenates were prepared using a lysis buffer. Cell lysates and tissue homogenates were prepared in lysis buffer (0.1 M Tris-HCl (pH 7.8), 0.1% Triton X-100, and 2 mM EDTA). The luciferase activity of each muscle group was determined. Similarly, a mixture of pDNA (pcDNA3-Luc; 30 mg/15 mL) and NBs (10 mg/10 mL) was intramuscularly administered into the tibialis muscle of ICR mice (5–6 weeks old, male) who were immediately treated with US (frequency, 1 MHz; duty, 50%; intensity, 2 W/cm^2^; time, 120 s). The tibialis muscle was harvested five days post-injection, followed by the estimation of luciferase activity.

### 2.5. In Vivo PMO Delivery into the Muscles of Mdx Mice Treated with NBs and US Exposure

Briefly, mdx mice (5–6 weeks old, male) were anesthetized, followed by hair removal at the predetermined site. Prior to each injection, a tourniquet was placed around the upper hind limb to restrict blood flow across the mouse limb. Subsequently, a mixture of the PMO (5 μg/15 mL) and NBs (10 mg/10 mL) was administered intravenously using a syringe pump (KDS 100: KD Scientific Inc., Holliston, MA, USA) at a constant flow rate (25 mL/144 s) via the great saphenous vein. Immediately after the injection, US (frequency, 1 MHz; duty, 50%; intensity, 2 W/cm^2^; time, 120 s) was applied to the hamstring muscle. Two weeks post-injection, the tibialis muscle in the US-exposed area was harvested, embedded in OCT compound, and immediately frozen at −80 °C. Then, specimens were analyzed using immunohistochemistry.

### 2.6. Immunohistochemistry

Briefly, serial sections were cut from treated and control muscles. The sections were stained with an NCL-DYS2 monoclonal antibody (Novocastra Lab. Co., Ltd., Newcastle upon Tyne, UK) known to react strongly with the C-terminal region of dystrophin. Alexa Fluor 546 (Thermo Fisher Scientific Inc., MA, USA) was used as the secondary antibody. Sections were examined under a fluorescence microscope (BZ8100; KEYENCE, Osaka, Japan). To enumerate dystrophin-positive fibers, the maximum number of dystrophin-positive fibers in each section was counted manually using analysis software for a fluorescence microscope, BZ Series (XG VisionEditor Ver.5.1). Muscle fibers were considered dystrophin positive if more than two-thirds of a single fiber exhibited continuous staining [31]. All positive fibers were counted in 3000 μm coronal sections on the knee side of each group (*n* = 4) and the mean value was calculated.

### 2.7. Evans Blue Dye Uptake

The Evans blue dye uptake study was performed as previously reported [32]. Briefly, PMO-treated mdx mice were exercised at 12 m/min for 30 min using the running system on a treadmill (MK-680C; MUROMACHI KIKAI Co., Ltd., Tokyo, Japan). An electric shock bar grid at the end of the tread delivered a mild shock when the mouse stopped running. Thirty minutes post-exercise, a solution of 5 μg/μL Evans blue dye (FUJIFILM Wako Pure Chemical Co., Ltd., Osaka, Japan) in saline was injected through the tail vein. The injection volume for each mouse was determined based on the individual body weight: 50 μL/10 g body weight. Twenty-four hours after injecting Evans blue dye, the mice were sacrificed, and the tibialis anterior and gastrocnemius muscles were dissected. The muscles were incubated overnight at 55 °C in formamide (5 L/mg body weight), and the supernatants were quantitated spectrophotometrically at 620 nm, as previously reported [33].

### 2.8. Statistical Analyses

All data are presented as mean ± standard deviation (SD; *n* = 4–6). Data were considered statistically significant at *p* < 0.05. The *t*-test was used to calculate statistical significance.

## 3. Results and Discussion

### 3.1. Effect of US Intensity on Luciferase Expression Following Treatment with NBs and US

**Figure 1:**
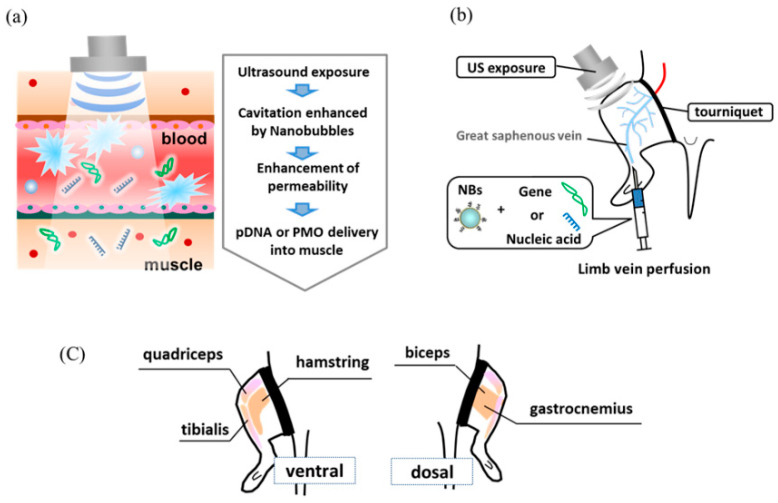
(**a**) Schematic diagram illustrating that in vivo administration of nanobubbles and US enhances tissue permeability. (**b**) A gene or nucleic acid delivery system for skeletal muscle administration via limb perfusion using nanobubbles and ultrasound. (**c**) Schematic diagram illustrating limb muscles of mice. NBs, nanobubbles; US, ultrasound.

**Figure 2:**
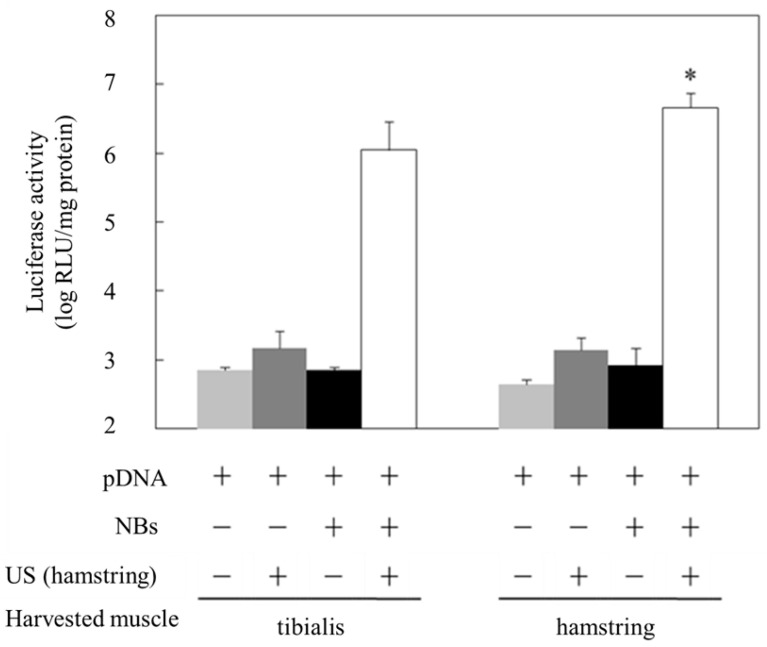
Luciferase expression in muscles transfected with NBs and US. US (frequency: 1 MHz, duty: 50%, intensity: 2 W/cm^2^, time: 2 min, exposure site: hamstring) was applied after injecting the solution (pDNA: 30 mg/15 mL, BL: 10 mg/10 mL). Muscles were harvested five days post-injection, and luciferase activity in the muscle was analyzed. * *p* < 0.05, compared with other groups. NBs, nanobubbles; pDNA, plasmid DNA; RLU, relative luminometer units; US, ultrasound.

**Figure 3:**
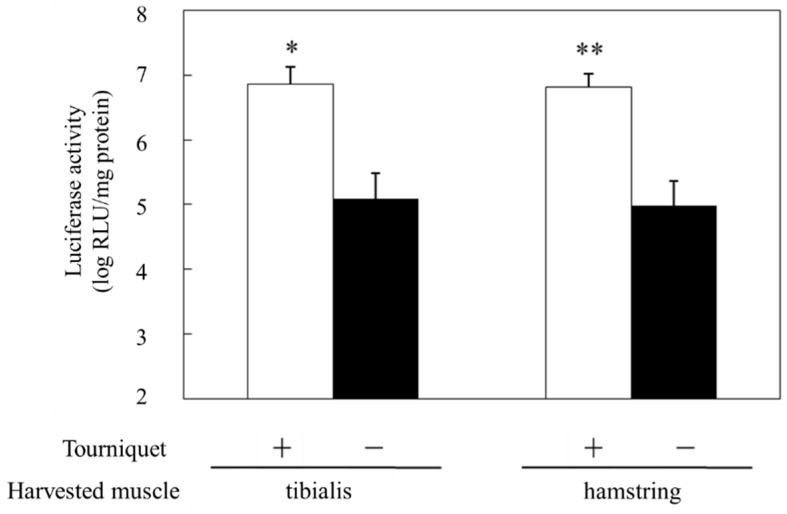
Effect of tourniquet on luciferase expression following treatment with NBs and US. US (frequency: 1 MHz, Duty: 50%, intensity: 2 W/cm^2^, time: 2 min, exposure site: hamstring) was applied after injecting the solution (pDNA: 30 mg/15 mL, BL: 10 mg/10 mL). Muscles were harvested five days post-injection, and luciferase activity in the muscle was analyzed. * *p* < 0.05, ** *p* < 0.005 compared with the negative control. NBs, nanobubbles; pDNA, plasmid DNA; RLU, relative luminometer units; US, ultrasound.

Previously, we have reported that the effect of gene transfer is locally enhanced when a mixture of NBs and pDNA encoding the luciferase gene is administered intramuscularly and accompanied by subsequent US exposure [26]. In addition, we demonstrated that the combined application of NBs and US exposure could achieve higher intramuscular PMO delivery than PMO injection alone, affording markedly enhanced dystrophin expression [28]. Considering muscle tissue transduction, for administering adeno-associated virus (AAV) via the limb vein, application of a tourniquet temporarily restricts blood flow and allows AAV accumulation, thereby affording efficient introduction into the muscle tissue [34]. Regarding the combined method using NBs and US, we hypothesized that applying a tourniquet to the upper part of the lower limb before limb vein perfusion with NBs and pDNA could allow widespread distribution into limb muscle tissues (Figure 1). To prevent solution leakage, we first applied a tourniquet around the upper part of the lower limb of a mouse. We then administered a mixture of NBs and pDNA via the great saphenous vein, followed by US exposure to the hamstring muscle, the location of the injection site. Only combined treatment with NBs and US exposure resulted in high luciferase activity in the hamstring muscle and near the tibialis anterior muscle (Figure 2). Next, we examined the effect of tourniquet application on gene transfection efficiency. Luciferase activity was significantly reduced in the group without tourniquet application, indicating the effectiveness of tourniquet application (Figure 3). Accordingly, the efficiency of the intravascular gene transfer could be explained by the restricted leakage of the perfusion solution (administered via limb vein) from the lower legs to the whole body.

### 3.2. Effect of US Intensity on Luciferase Expression Following Treatment with NBs and US

**Figure 4:**
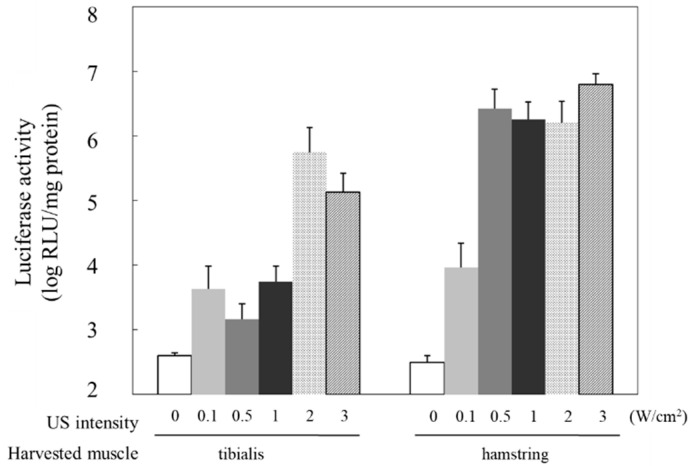
Effect of US intensity on luciferase expression following treatment with NBs and US. US (frequency: 1 MHz, duty: 50%, intensity: 0–3 W/cm^2^, time: 120 s, exposure site: hamstring) was applied after injecting the solution (pDNA: 30 mg/15 mL, BL: 10 mg/10 mL). Muscles were harvested five days post-injection, and luciferase activity in the muscle was analyzed. NBs, nanobubbles; pDNA, plasmid DNA; RLU, relative luminometer units; US, ultrasound.

Considering gene transfer using US, it has been reported that US exposure conditions, such as intensity, can impact gene transfer efficiency [17,35]. Therefore, we examined the effect of US exposure on the efficiency of gene transfer administered via the limb vein. On applying an intensity ranging between 0 and 3 W/cm^2^, we noted an intensity-dependent increase in the activity between 0 and 0.5 W/cm^2^, which plateaued at ≥0.5 W/cm^2^ in the hamstring muscle (Figure 4). The tibialis anterior muscle, located at a distance from the US exposure site, exhibited higher luciferase activity than the other muscles at >2 W/cm^2^ (Figure 4).

### 3.3. Comparison of Intravenous and Intramuscular Gene Delivery

**Figure 5:**
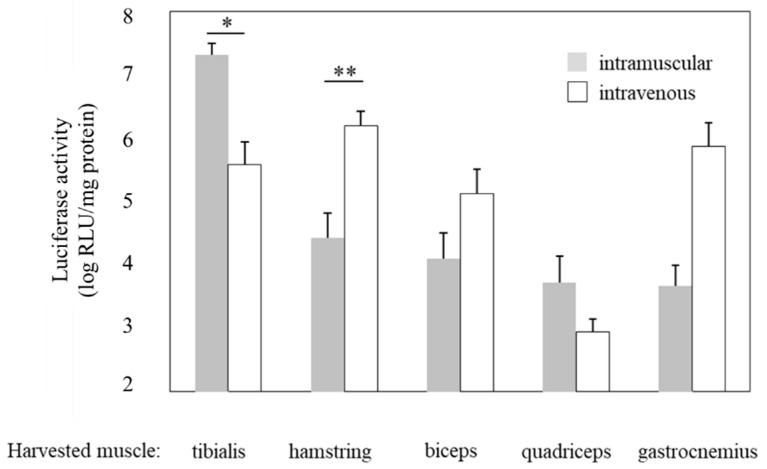
Comparison between intramuscular and intravenous injections following treatment with NBs and US. US (frequency:1 MHz, duty: 50%, intensity: 2 W/cm^2^, Time:120 s, exposure site: hamstring (intravenous) or tibialis (intramuscular)) was applied after injection of the solution (pDNA:30 mg/15 mL, BL:10 mg/10 mL). Muscles (tibialis, hamstring, biceps, quadriceps, and gastrocnemius) were harvested five days post-injection, and luciferase activity in the muscles was analyzed. * *p* < 0.01, ** *p* < 0.05. NBs, nanobubbles; pDNA, plasmid DNA; RLU, relative luminometer units; US, ultrasound.

**Figure 6:**
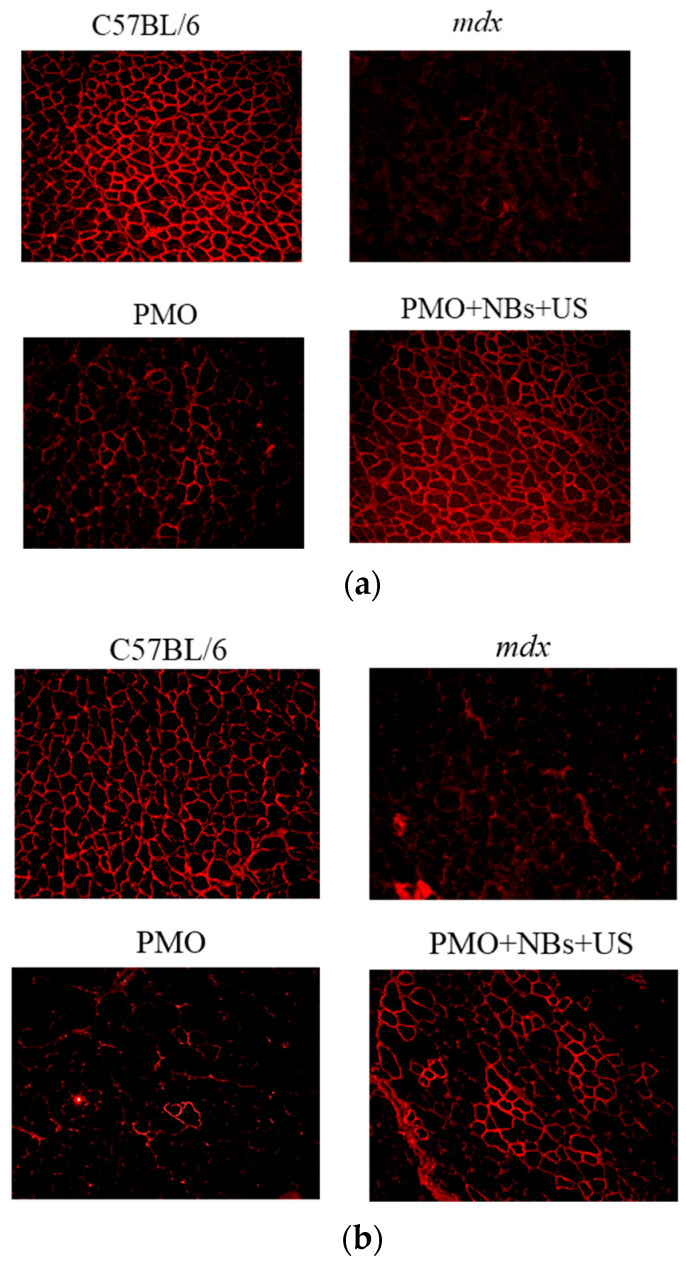
Detection of dystrophin expression in muscles using immunohistochemistry. Dystrophin expression in limb muscles was detected by immunohistochemistry two weeks after intravenously administering the PMO (50 mg/15 mL) with bubble liposomes (10 mg/10 mL) and ultrasound (frequency: 1 MHz, duty: 50%, intensity: 2 W/cm², time: 2 min). Dystrophin expression was examined under a fluorescence microscope. Normal C57BL/6 mice served as positive controls. (**a**) Hamstring muscle. (**b**) Gastrocnemius muscle. Magnification: 100×. NBs, nanobubbles; PMO, phosphorodiamidate morpholino oligomer; US, ultrasound.

Compared with gene transfer administered intramuscularly, which is widely used as a simple method for gene transfer to skeletal muscle in vivo, perfusion via the limb vein could afford gene delivery to a wider range of muscle tissues [36,37]. Therefore, we compared the gene transfection efficiency in different muscle tissues of the group administered a pDNA and NB mixture intramuscularly into the tibialis anterior muscle followed by US exposure and the group administered pDNA and NBs intravenously via limb vein perfusion followed by US exposure to the hamstring muscle. The intramuscular group showed high luciferase activity only in the tibialis anterior muscle at the injection site, whereas the intravenous group exhibited sufficient activity in a wide area of muscle tissues (Figure 5). Accordingly, these results indicated that using a tourniquet to accumulate NBs and pDNA in the blood vessels dominating the muscle tissue is a more effective strategy than local administration. As shown in Figure 6, activity in a wide area of muscles was only observed in the pDNA + NBs + US treatment group. This finding may be due to cavitation induction followed by the destruction of NBs after US exposure occurring over a large muscle area.

### 3.4. Intravenous PMO Delivery into the Muscle of DMD Model Mice

**Figure 7:**
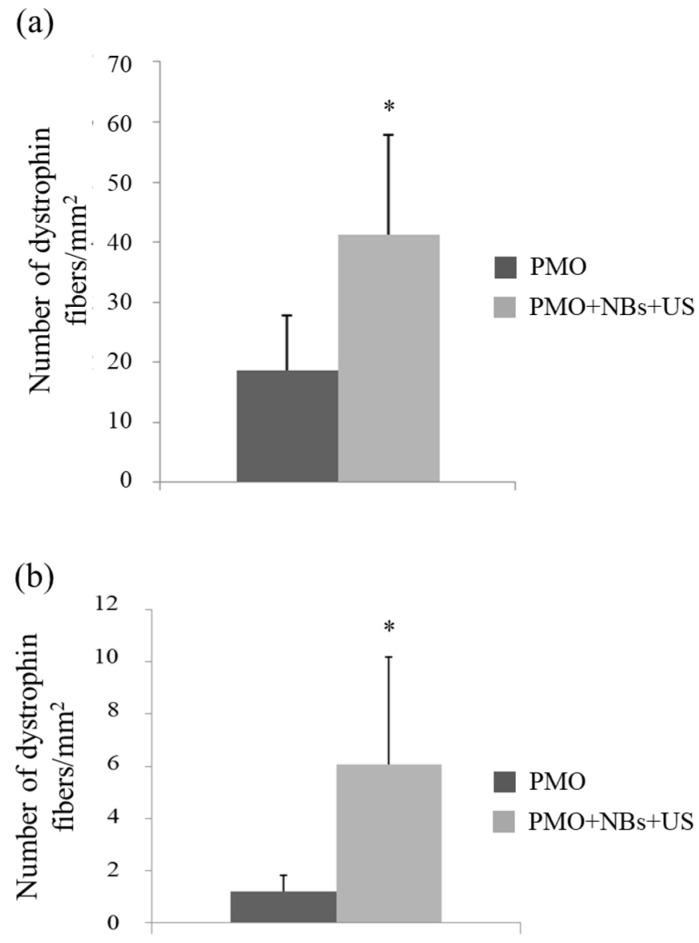
Number of dystrophin-positive fibers per unit area. The number of dystrophin-positive fibers was measured using a fluorescence microscope. (**a**) Hamstring and (**b**) gastrocnemius muscles. * *p* < 0.05. NBs, nanobubbles; PMO, phosphorodiamidate morpholino oligomer; US, ultrasound.

To assess whether the intravascular delivery method using a combination of NBs and US exposure is beneficial for treating muscle diseases, we attempted to deliver a PMO that targeted the mutated mouse dystrophin exon 23 and enabled the recovery of dystrophin expression into *mdx* mice, used as a DMD model [6,9]. First, after applying a tourniquet to the upper part of the lower limb, the fluorescently labeled PMO and NBs were injected intravenously via limb perfusion into the *mdx* mice, followed by immediate US exposure. Using the described method, we achieved treatment delivery to a significantly high number of myofibers in a wide area of the hind limb muscles (quadriceps, biceps, hamstring, gastrocnemius, and tibialis). Next, the PMO was delivered to the limb muscles of mdx mice using a similar method. Two weeks later, the exon-skipping levels in the treated muscles were analyzed using immunohistochemistry. The NB and US exposure group exhibited a significantly higher number of dystrophin-positive fibers in hamstring and gastrocnemius muscle cross-sections than the PMO-alone group (Figure 6a,b), along with an enhanced number of positive fibers per area (Figure 7a,b).

**Figure 8:**
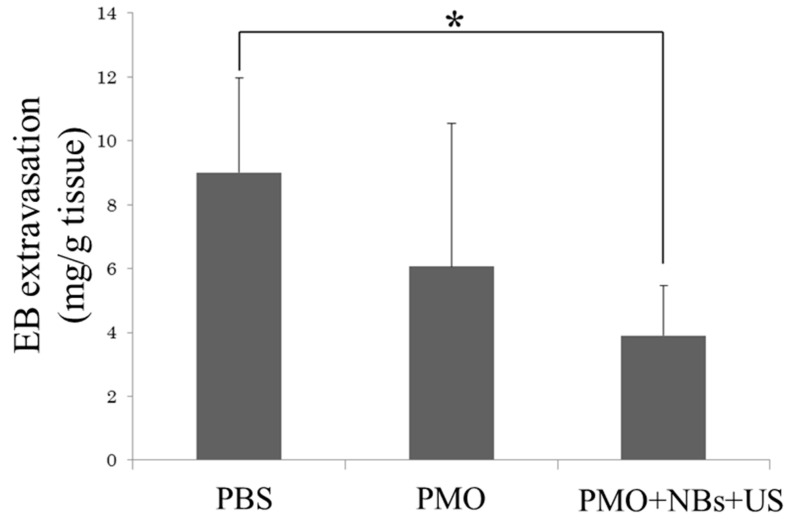
Therapeutic effect of PMO on hamstring muscles along with NBs and US using the Evans blue permeability assay. US (frequency, 1 MHz, Duty, 50%; intensity, 2 W/cm²; time, 2 min) after injecting PMO (50 mg/15 mL) with NBs (10 mg/10 mL). The mdx mice were exercised for 30 min on a treadmill (MK-680S), and Evans blue was administered via the mouse tail vein. After 24 h, hamstring muscles were extracted using formamide. Subsequently, the Evans blue permeation was determined spectrophotometrically. * *p* < 0.05. EB, Evans blue; NBs, nanobubbles; PMO, phosphorodiamidate morpholino oligomer; US, ultrasound.

Evans blue dye is a membrane-impermeable dye that binds to serum albumin; however, disruption of the plasma membrane allows dye entry into the cell [38,39]. Thus, the uptake of Evans blue dye by muscle fibers indicates the presence of membrane damage in the muscle fibers. Therefore, we evaluated the effect of the PMO treatment on muscle cell membrane strength in mice with dystrophin protein deficiency. Two weeks after the intravascular PMO delivery using a combination of NBs and US exposure, the PMO-treated mdx mice were subjected to treadmill running, followed by tail vein injection of Evans blue dye; subsequently, we evaluated dye uptake into the muscles. The combined NBs and US group exhibited less dye permeation than the PMO-alone group (Figure 8a,b); this finding may be due to the suppression of cell membrane permeability by sarcolemma repair, with an increase in dystrophin protein by PMO delivery via NBs and US exposure.

These results suggest that intravascular delivery via limb vein perfusion combined with NBs and US exposure may be useful for delivering pDNA or PMOs to a wide range of muscle cells. The exon-skipping method using ASOs is a revolutionary technology to achieve feasible therapeutic strategies, and recently, several FDA-approved ASOs (Exondys 51, Vyondys 53, and Viltepso Amondys 45) have been clinically applied for the exon skipping of DMD genes [10]. However, the therapeutic efficacy of ASOs is temporary, necessitating lifelong administration. Nevertheless, genome editing tools (e.g., CRISPR-Cas systems) have markedly advanced mammalian genome editing owing to their high efficiency and methodological simplicity [40,41]. Recent studies in mice and dogs using AAVs expressing the CRISPR/Cas9 system have reported the correction of mutated exons in DMD [42,43,44]. Viral vectors such as AAV, lentivirus, and adenovirus are still widely employed as delivery methods for mammalian gene editing given their high efficiency; however, viral vectors present challenges, such as a strong immune response, insertional mutagenesis, and other safety issues [45]. These disadvantages of viral vectors have restricted advanced research on gene editing using CRISPR/Cas9. Therefore, the development of nonviral delivery methods for CRISPR/Cas9 is required. Accordingly, combining NBs and US may be valuable for improving the delivery efficiency of ASOs, including PMOs, as well as for delivering CRISPR/Cas systems for future application in DMD therapy [46].

In addition, this delivery method for muscle tissues may be a potential tool for the treatment of diseases other than DMD, by introducing different genes and nucleic acids into various muscle tissues. For example, studies have reported that exogenous functional genes, such as those producing antibodies for cancer immunotherapy [47] and insulin analogs for diabetes treatment [48], are injected intramuscularly to supply the produced proteins to the entire body, thereby treating these diseases. Using NBs and US, pDNA that encodes secreted proteins can be introduced into a wide range of muscle tissues through intravenous limb injections, which may further improve therapeutic efficacy. This approach holds promise for application in various diseases in which secreted proteins are anticipated, including rheumatoid arthritis, vascular disorders with systemic symptoms, cancer, and diabetes.

Recently, RNA-based therapeutics have gained significant attention as powerful platforms for the treatment of various diseases. Nucleic acids other than ASOs, such as small interfering RNA (siRNA) and microRNA (miRNA), exert their therapeutic effects by regulating the expression patterns of disease-related genes and are applicable to the treatment of diverse diseases [10]. Since the approval of the first siRNA drug by the FDA in 2018, the number of approved sequences has increased and clinical trials have progressed. With the approval of mRNA-based vaccines in 2020, the effective use of mRNA as a protein replacement therapy to produce therapeutic proteins has attracted significant attention. In this context, the stabilization of RNA-based molecules by chemical modification and the application of efficient intracellular delivery systems, including lipid nanoparticles (LNPs), are becoming increasingly important to ensure that RNA-based molecules are delivered to target sites without degradation in the body [10]. Indeed, when a mixture of pDNA encoding the luciferase gene, its gene-specific siRNA, and NBs was introduced into a wide range of muscle tissues through intravenous limb injection in normal mice followed by immediate US exposure, the results showed that approximately 80% inhibition of luciferase activity was observed in a wide range of muscle tissues. Although a transient gene transfer experiment was performed, this method using NBs and US suggests that the delivery of siRNA to muscle tissue is feasible. In addition, we previously reported that plasmid DNA or nucleic acids can be loaded onto the surface of NBs containing cationic lipids by electrostatic interactions and that nucleic acid delivery is possible in combination with US exposure [49,50,51]. We further created NBs loaded with microRNA-126, a known angiogenic factor, and evaluated their therapeutic potential when systemically administered to a mouse model of hindlimb ischemia; the results showed several angiogenic factors were increased and blood flow was significantly improved. Nucleic-acid-loaded NBs were significantly more effective than a mixed solution of free nucleic acids and neutral NBs, suggesting that molecule-loaded NBs have a greater therapeutic value [50]. For these reasons, by using NB and US, RNA-based therapeutics can be also delivered into a wide range of muscle tissues via intravenous limb injection. This enables the treatment of diseases that specifically target muscle tissues, including neuromuscular diseases such as DMD. Additionally, it may be possible for treating systemic diseases through mRNA-based protein replacement therapy, such as the application of plasmid DNA, as well as for regenerative medicine.

Skeletal muscles typically account for approximately 30% of an adult’s body weight, with the muscles of the lower extremities accounting for a large proportion of that weight. Therefore, it is considered an excellent target tissue for improving the pathophysiology of genetic diseases, such as muscular dystrophy and other disorders, through gene therapy. Skeletal muscle has a large number of blood vessels; therefore, it is also possible to treat other systemic diseases by delivering locally expressed proteins throughout the body. Therefore, the intravascular delivery of therapeutic genes and nucleic acids into a wide range of muscle tissues via limb vein perfusion by the combination of NBs and US exposure is of great practical value. In particular, when gene therapy is applied to diseases such as muscular dystrophy, which lead to degeneration and atrophy in skeletal muscles throughout the body, it is desirable to achieve transgene expression in muscle tissues throughout the body. Therefore, the development of gene transfer technology targeting a wide range of tissues, rather than just local ones, is desired. For these reasons, to extravasate through the barrier between blood vessels and muscle cells by the combination of NBs and US, pDNA and PMOs were transfected into a wide range of tissues in the lower limbs of mice via the blood vessels surrounding the muscle tissue.

It has been reported that local injection of substantial amounts of DNA (through the hydrodynamic injection method) can lead to significant levels of gene expression in limb muscles. With the inflow and outflow of blood blocked surgically or with an external tourniquet, large amounts of naked DNA can be delivered locally into muscle cells by hydrodynamic intravascular administration [52]. Compared with the hydrodynamic limb vein injection methods (e.g., 1 mL/leg of mouse) [53], our injection volume (25 µL/leg/25 g of mouse) was smaller. Smaller doses may avoid risks such as compartment syndrome [54]. In contrast to our strategy, when considering the intravascular administration of mRNA using this method, mRNA is degraded by nucleases in the blood. However, combining this NB and US method with lipid nanoparticles or cationic polymers that protect mRNA from degradation will enable efficient and low-dose delivery of mRNA into the muscles, even by intravenous injection. In this way, while specifically delivering mRNA as well as other genetic material to affected areas of various diseases, the unnecessary uptake of nanoparticles and their materials may be avoided, thereby eliminating off-target effects. In addition, since NBs can function as an US contrast agent, the process of NBs and therapeutic drug administration can be confirmed while US imaging is performed with an US diagnostic device to complete the treatment. Thus, it would be easy to construct a theranostic system [51].

Nevertheless, there are still concerns and considerations to be resolved before applying NBs to clinical practice. Due to concerns over US-associated tissue heating due to increased frequency, US intensity and its exposure time should be carefully considered. Depending on the intensity and duration of the ultrasound, a temperature rise may occur, leading to tissue damage. Therefore, US intensities in the range of 0.3 and 3 W/cm^2^ are commonly used for drug or gene delivery to avoid heating the US-irradiated site [55]. Therefore, we employed similar US intensities in the range of 0.3 and 3 W/cm^2^. As shown in Figure 4, on applying an US intensity ranging between 0 and 3 W/cm^2^, the tibialis anterior muscle, located at a distance from the hamstring muscle that was the US exposure site, exhibited higher luciferase activity than the other muscles at >2 W/cm^2^ (Figure 4). No temperature increase was observed with this US exposure. In addition, the luciferase activity plateaued at ≥0.5 W/cm^2^ in the hamstring muscle, the luciferase activity was at a low level in the tibialis anterior muscle between 0.5 and 1 W/cm^2^. As shown in Figure 5, the results indicated that using a tourniquet to accumulate NBs and pDNA in the blood vessels dominating the muscle tissue is a more effective strategy than local administration. However, the luciferase activity in part of limb muscle (biceps and quadriceps) was still at a low level. This was due to the use of a 6 mm-diameter US probe, which limited the transmission range of the ultrasound. Therefore, the use of wider US probes may offer more potential to safely and efficiently deliver genes and nucleic acids into a broad range of muscle tissues without increasing the US intensity. Furthermore, establishment of a gene and nucleic acid delivery method using a scanner probe for human US diagnosis is expected to increase its practical value. However, long-term or repeated application of US for targeted MB disruption has been reported to be associated with damage such as microvascular integrity and hemolysis [20,56]. Therefore, we believe that future research on methods to deliver NBs and US exposure to muscle tissue should include detailed experiments on the toxicity, inflammatory response, hemolysis, and vascular damage associated with cavitation induction, as well as the side effects associated with repeated administration. The information obtained from these experimental data should be used to optimize this delivery method.

In conclusion, we developed a novel method for gene and nucleic acid delivery into skeletal muscle via limb perfusion using NBs and US. Indeed, compared with gene transfer administered intramuscularly, which is widely used as a simple method for gene transfer to skeletal muscle in vivo, perfusion via the limb vein enabled afford gene delivery to a wider range of muscle tissues. Furthermore, we successfully established this combination method for delivering PMOs intravenously to a wide range of skeletal muscles in DMD model mice and demonstrated the increase in the number of dystrophin-positive fibers. In contrast, ASOs currently in clinical application are administered intravenously once a week and nearly 99% of the dose is rapidly excreted by the kidneys without transfer to muscle tissue [11,12]. Therefore, utilizing NB and US techniques for ASO administration would not only provide efficient therapeutic effects with smaller doses but would also reduce the economic burden. Although the symptoms of DMD patients are not limited to the muscles of the lower limbs, the development of a therapeutic US device capable of irradiating the entire body will make it possible to treat the skeletal muscles of the entire body in the future. Thus, this US-mediated NB technique may provide an effective, non-invasive, and non-viral method for ASO therapy for DMD muscles, as well as for other forms of muscular dystrophy. Although there are still many issues to be resolved in the clinical application, the combination of NBs and US, which can widely expose the hindlimb muscles via extremity veins, may be a feasible method in gene and nucleic acid therapies for DMD and other neuromuscular diseases, as well as other non-neuromuscular diseases.

## Data Availability

Not applicable.
